# The southward expansion of *Anopheles farauti* (Diptera: Culicidae) in Australia: a genetic and ecological niche modeling investigation

**DOI:** 10.1093/jme/tjag158

**Published:** 2026-10-20

**Authors:** Fitsum Bekele, Luke Ambrose, Andrew F van den Hurk, Cassie C Jansen, Weng K Chow, Meredith Larney, Tristan P W Dennis, Nigel W Beebe

**Affiliations:** School of the Environment, The University of Queensland, Brisbane, Australia; Australian Defence Force Malaria and Infectious Disease Institute, Gallipoli Barracks, Enoggera, Brisbane, Australia; Public and Environmental Health Reference Laboratories, Queensland Health, Brisbane, Australia; Health Protection and Regulation Branch, Queensland Health, Brisbane, Australia; Australian Defence Force Malaria and Infectious Disease Institute, Gallipoli Barracks, Enoggera, Brisbane, Australia; Central Queensland Public Health Unit, Queensland Health, Rockhampton, Australia; School of the Environment, The University of Queensland, Brisbane, Australia; Department of Vector Biology, Liverpool School of Tropical Medicine, Liverpool, UK; School of the Environment, The University of Queensland, Brisbane, Australia

**Keywords:** *Anopheles farauti*, ecological niche modeling, population genetics, malaria vector

## Abstract

The mosquito *Anopheles farauti* Laveran (1902) is a coastal malaria vector with a wide distribution throughout the Southwest Pacific. This species shows strong population genetic structure across Australia, New Guinea, and the Solomon Archipelago. In Queensland, Australia, its southern range limit has been the 21° latitude but, in 2023, it was detected in mosquito surveys 300 km further south, at 23° latitude. Additional collections undertaken in 2024 suggested that a population of *An. farauti* had established at this new southern limit. Sequencing of the mitochondrial cytochrome oxidase I (COI) gene revealed a single haplotype shared by all individuals, matching the nearest northern population. To investigate the suspected southward range expansion of *An. farauti* in Queensland, we performed machine learning ecological niche modeling using both MaxEnt and Random Forest approaches using historical collection data and WorldClim bioclimatic variables from that period. The model output indicated that the 23° latitude in Queensland is within the species’ projected suitability range and identified temperature-related factors as the primary drivers of its distribution in Queensland. We hypothesize that the prolonged La Niña (2020 to 2023), and the 2023 global marine heatwave, which drove warmer ocean and coastal ambient temperatures, facilitated the latitudinal expansion of *An. farauti* in Queensland.

## Introduction

The mosquito, *Anopheles farauti* Laveran (1902), is a key member of the *Anopheles punctulatus* group, and is responsible for much of the malaria burden in the Southwest Pacific (Cooper et al. 2009, Beebe et al. 2015). This species is morphologically indistinguishable from its closest relatives and inhabits the broadest geographical range within the group. It occurs from the Indonesian Moluccas in the west, across New Guinea and the Solomon Islands, extending south to Vanuatu and northern Australia (Foley et al. 1993, 1994, Cooper et al. 1995, 1996, 1997, 2002, 2009). *Annopheles farauti* is largely restricted to coastal areas, and its larvae can tolerate saline conditions and develop in both brackish and freshwater habitats (Sweeney 1987, Beebe and Cooper 2002). These habitats include coastal lagoons and swamps, which are often close to human settlements. Together with its ability to feed on both humans and animals, this habitat preference contributes to its importance as a malaria vector in coastal regions of Papua New Guinea (PNG) and the Solomon Archipelago (Bugoro et al. 2011, Keven et al. 2022).

Across this vast region of the Southwest Pacific, *An. farauti* shows striking population genetic and geographic structure. Studies using microsatellite, nuclear and mitochondrial DNA (mtDNA) revealed 3 main genetic groups (Ambrose et al. 2012, 2014, 2024). One group encompasses northern Australia, the Torres Strait Islands (a group of islands between northeastern Australia and southern New Guinea), and southern New Guinea. Another comprises populations across the rest of New Guinea (including the Papuan Peninsula and northern areas), and a third spans the islands of the Solomon Archipelago, extending south to the remote islands of Vanuatu. There is additional population genetic structure within each of these 3 main groups (Ambrose et al. 2012, 2014, 2024).

The southern limit of *An. farauti* in Australia was initially thought to be 19°S, near Townsville (Fig. 1) and formed the accepted southern limit of Australia’s malaria receptive zone (Ford 1950). In the 1990s, a population was detected in wetlands in and around Mackay, ∼350 km south of Townsville (van den Hurk et al. 1998). Subsequent mosquito surveys, including those by the Australian Defence Force (ADF) at their Shoalwater Bay Training area approximately 150 km south of Mackay, between March 1998 and February 2000 did not collect *An. farauti* (Frances et al. 2004).

**Fig. 1.**
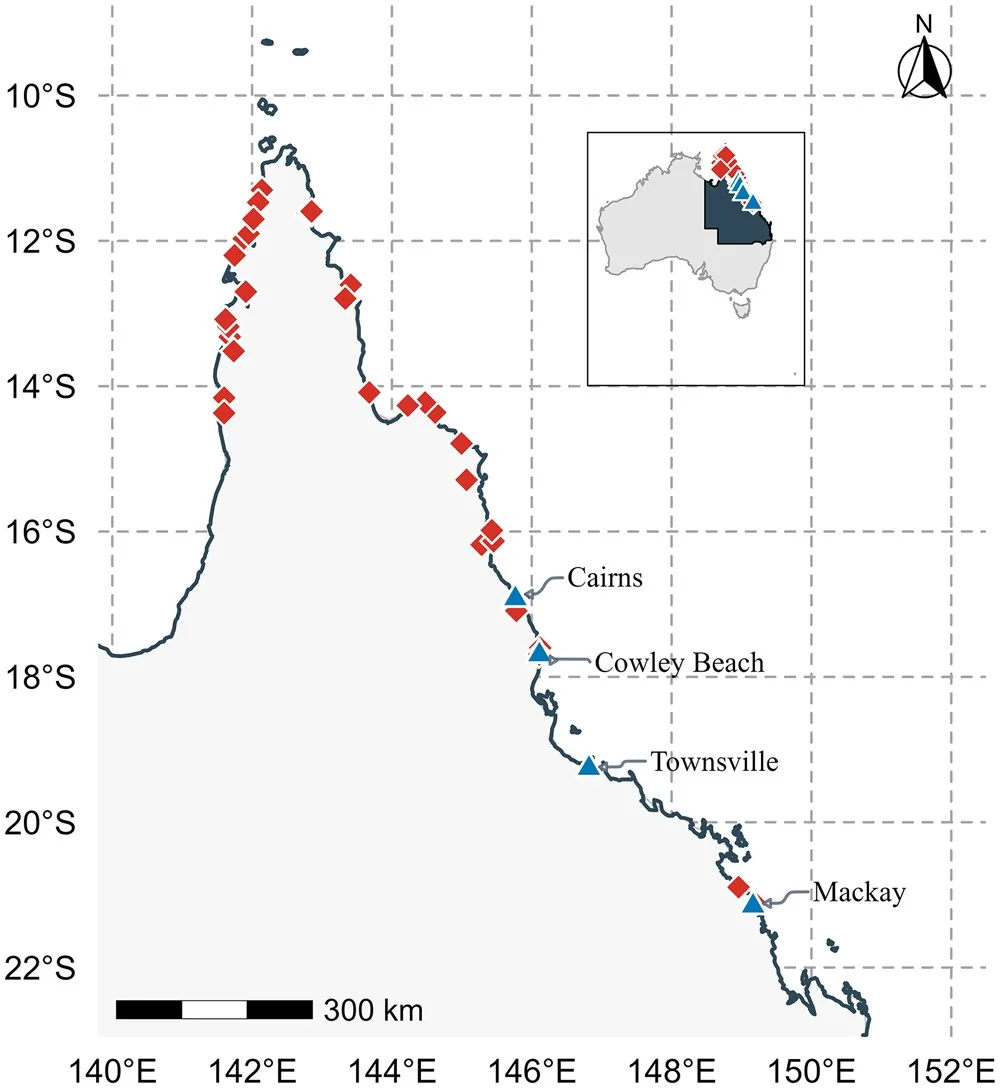
Spatial distribution of *An. farauti* sampling sites used in the ecological niche modeling. Diamonds represent the sampling sites, and triangles indicate key coastal localities along the Queensland coastline.

In 2023, a member of the *An. farauti* species complex was identified in mosquito collections from the Kinka Wetlands near Rockhampton, 300 km south of Mackay (Fig. 1). As *An. farauti* is morphologically indistinguishable from *Anopheles hinesorum* Schmidt (2001) and *Anopheles torresiensis* Schmidt (2001) (formerly called *An. farauti* 2 and 3, respectively), molecular analysis was used to confirm that the specimen collected in the Kinka Wetlands was *An. farauti* (van den Hurk et al. 2024). This study aims to determine the geographical origin of the population of *An. farauti* collected from the Kinka Wetlands of Central Queensland. We hypothesize that the incursion is a latitudinal shift of the southern (Queensland) population of *An. farauti* that would be consistent with a warming climate. We genetically confirm the population’s origin and develop an ecological niche model of this genetically and geographically restricted Queensland population. We utilize historical and contemporary sampling sites to estimate the habitat suitability range for *An. farauti* and identify the key environmental factors influencing its distribution. While the Australian mainland remains free of local malaria transmission, understanding the habitat preferences of this mosquito in Queensland offers insights into its ecology, informing surveillance efforts and the potential distribution of this malaria vector in a warmer climate change future.

## Methods

### Sample Collection

Following the initial detection of *An. farauti* in the Kinka Wetlands in 2023 (van den Hurk et al. 2024), additional, targeted mosquito samples were collected from Kinka Wetlands in Central Queensland (23°14′S, 150°46′E) in 2024. A total of 32 traps were deployed on 8 collecting trips undertaken between 29 February and 20 June 2024. Four traps were deployed each night with the frequency of trapping ranging from 1 to 3 weeks depending on staff availability and site accessibility. Traps were set at 5 sites in the Kinka Wetlands and at 7 other sites within 5 km of the Kinka Wetlands. The original trap site in the Kinka Wetlands where *An. farauti* was discovered in 2023 was sampled on 8 occasions, whilst other sites were sampled from 1 to 6 times. Mosquitoes were collected using CO_2_-baited PB light traps (Pacific Biologics, Kippa Ring, Australia) deployed overnight. Collected specimens were transported on dry ice to the Public and Environmental Health Reference Laboratories, Brisbane, where mosquitoes in the *An. farauti* complex were morphologically identified using the dichotomous key of Lee et al. (1987). Specimens identified as members of the *An. farauti* complex were forwarded to the Vector Biology Laboratory at The University of Queensland for molecular analysis.

### Molecular Identification of Cryptic Species and Regional Genotype

Genomic DNA was extracted from samples according to the protocol of Beebe et al. (1994). Ribosomal DNA (rDNA) internal transcribed spacer 2 (ITS2) was amplified to identify species status (Beebe and Saul 1995) and ITS1 genotyping was used to confirm the regional genotype (Beebe et al. 2000).

### Sequence Analysis of Mitochondrial DNA Cytochrome c Oxidase Subunit I

A portion of the mitochondrial cytochrome c oxidase subunit I (COI) gene was PCR amplified following the protocol described by Ambrose et al. (2012), and PCR products were Sanger sequenced by Macrogen Inc. (Seoul, Korea). Raw sequences were inspected and edited in Geneious Prime v2022.2.2 (Biomatters Ltd., Auckland, New Zealand). Following sequence quality assessment, usable COI sequences were obtained from 19 of the 24 *An. farauti* specimens collected at Kinka Wetlands in 2024. These sequences were initially compared with records in the NCBI GenBank database using BLAST to confirm sequence identity.

For population genetic comparison, 77 reference COI sequences were obtained from GenBank from the dataset of Ambrose et al. (2012), representing sampling sites in Cape York Peninsula, the Torres Strait Islands, and southern New Guinea (Table 1; Fig. 2). These reference sequences were included because the previous study (Ambrose et al. 2012) identified mitochondrial patterns associated with historical introgression with *An. hinesorum* and a mitochondrial selective sweep across Queensland and southern New Guinea.

**Table 1.** Sampling locations and population site codes used in to generate the COI haplotype network

Site	Region	Coordinates	
		Lat	Lon
**0f**	Queensland-Kinka Wetlands	−23.233	150.788
**1f**	Queensland-Mackay	−21.133	149.183
**2f**	Queensland-Cowley Beach	−17.691	146.107
**3f**	Queensland-Cape York Peninsula	−14.086	143.676
**4f**	Queensland-Cape York Peninsula	−10.778	142.415
**9f**	Queensland-Torres Strait	−10.167	142.200
**10f**	Indonesia-Timika	−4.835	136.758
**16f**	Papua New Guinea-southern New Guinea	−8.043	143.723

The 19 Kinka Wetlands sequences and 77 reference sequences were then aligned in Geneious Prime, producing a final 474-bp alignment of 96 sequences. Haplotype relationships were generated and visualized using PopArt v1.7 (Leigh and Bryant 2015), and population genetic parameters were calculated using the pegas package in R (Paradis 2010).

### Ecological Niche Modelling

#### Species Occurrence Records

The *An. farauti* occurrence data were obtained from previous mosquito surveys conducted by the ADF in far north Queensland at the end of the wet season (April-May) in 1985, 1986, and 1991 (Cooper et al. 1996) and our own collections in Central Queensland in regions between Townsville and Mackay in 2021 (unpublished data; Supplementary Table S1; Fig. 1). The historical ADF surveys contributed 40 occurrence locations, while the 2021 collection contributed 1 additional location, resulting in a total of 41 occurrence locations used for ecological niche modeling. All specimens morphologically identified as belonging to the *An. farauti* complex were identified to species by either allozyme electrophoresis (Mahon 1984), DNA probes (Cooper et al. 1991) or PCR (Beebe and Saul 1995).

#### Predictor Variable Selection

The initial predictor set included nineteen WorldClim bioclimatic variables (Supplementary Table S2) (Fick and Hijmans 2017), together with relative humidity and elevation. The predictor variable code, name and description are provided in Supplementary Table S2. Multicollinearity was assessed using pairwise Pearson correlations (|r| > 0.7; Dormann et al. 2013). Correlated variables were grouped, and within each group, the predictor most closely aligned with the life history and habitat preferences of *An. farauti* was retained (Cooper et al. 2002, Sweeney et al. 2006). Response curves from a preliminary MaxEnt run using all variables (Phillips et al. 2006) further informed the final variable selection.

#### Modeling

To explore the putative habitat of *An. farauti* in Queensland, we applied 2 correlative machine-learning approaches, MaxEnt (Phillips et al. 2006) and Random Forest (Cutler et al. 2007), using the EcoCommons platform (EcoCommons Australia 2024). Random Forest was included because it can capture complex, non-linear responses and interactions among predictors. MaxEnt, a presence-only method based on the principle of maximum entropy, is well suited to limited and presence-only datasets (Evans et al. 2011, Lissovsky and Dudov 2021).

Both models were initially fitted using the same 41 occurrence records (Supplementary Table S1) and environmental predictors at ∼1 km resolution (Supplementary Table S2). For exploratory purposes, background samples for the model were drawn from the full extent of Queensland. MaxEnt was run using platform defaults (automatic feature selection, regularization multiplier = 1, 10,000 background points, logistic output). For Random Forest, because true absence data were unavailable, EcoCommons generated random pseudo-absences (excluding known presences) at a 1:1 ratio, and models were fitted using default settings (500 trees, bootstrap resampling with replacement, and the square-root of the number of predictors tested at each split). Model fitting and evaluation employed 10 repetitions of spatially blocked cross-validation. Performance was assessed using the area under the receiver operating characteristic curve (AUC), and Random Forest variable importance was quantified using the mean decrease in Gini index.

Following this exploratory comparison, MaxEnt was retained for subsequent model development and refinement. To better represent the area accessible to the predominantly coastal *An. farauti* population, we defined an ecologically informed accessible area (M) following Barve et al. (2011), restricting background point selection to a 30 km coastal buffer. MaxEnt model complexity was then tuned following Merow et al. (2013) and Radosavljevic and Anderson (2014). Candidate models used linear, quadratic, and product (LQP) features with regularization multipliers (β) ranging from 1 to 5 and were evaluated using 10-fold cross-validation. The final model employed LQP features with β = 2.5.

To estimate prediction uncertainty, we generated 10 bootstrap replicates of this model. The mean output was used as the habitat suitability map, while the standard deviation across replicates provided an index of spatial uncertainty. Variable contributions were evaluated using permutation importance and jackknife tests. Suitability maps were processed and visualized in R (v4.1.2) using raster (Hijmans 2024), ggplot2 (Wickham 2016), cowplot (Wilke 2025), and dplyr (Wickham et al. 2025) to standardize color scales and improve figure presentation.

## Results

### Molecular Identification of Cryptic Species and Regional Genotype

A total of 24 mosquitoes morphologically identified as belonging to the *An. farauti* complex were collected in 8 of the 32 traps deployed at Kinka Wetlands. Single specimens were collected in 2 traps deployed at the original site in the Kinka Wetlands, whilst traps set at 5 other sites yielded from 1 to 13 *An. farauti* complex mosquitoes. All specimens were confirmed to be *An. farauti* by *Msp*I restriction digest of the rDNA ITS2 (Fig. 3). As regional genotypes of *An. farauti* can be identified by *Msp*I restriction digestion of the rDNA ITS1 region and gel electrophoresis (Beebe et al. 2000), the Kinka Wetland samples (Fig. 3, samples 1 to 3) displayed restriction bands at approximately 480, 380, 180, and 150 bp, consistent with the *An. farauti sensu stricto* genotype commonly found in Queensland populations. These profiles were identical to those observed in Queensland reference samples from Cowley Beach south of Cairns (samples 4 and 5). In contrast, a genotype profile of a sample of *An. farauti* from a PNG Rabaul colony showed a distinct banding profile of the PNG genotype, with major fragments at approximately 580, 350, 150, and 100 bp (sample 6, Fig. 3).

**Fig. 2.**
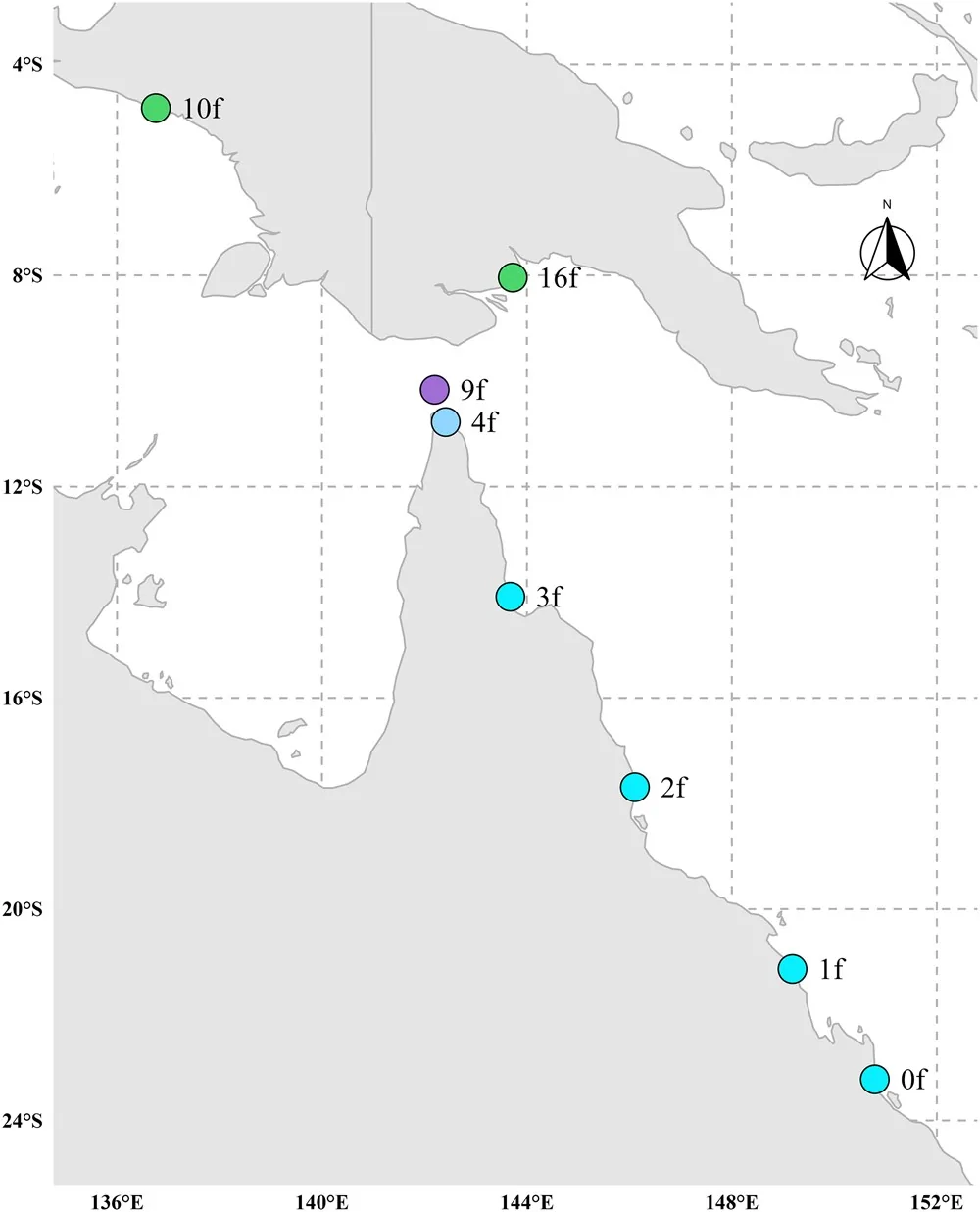
Sampling locations and corresponding site codes used in the haplotype network analysis of the 96 *An. farauti* mitochondrial COI sequences, including sequences from Ambrose et al. (2012). Sampling sites are detailed in Table 1 and include “0f” (Kinka Wetlands). Sites 1f from Mackay, 2f from Cowley Beach Training Area with 3f and 4f from Cape York (site numbering the same as in Ambrose et al. 2012).

**Fig. 3.**
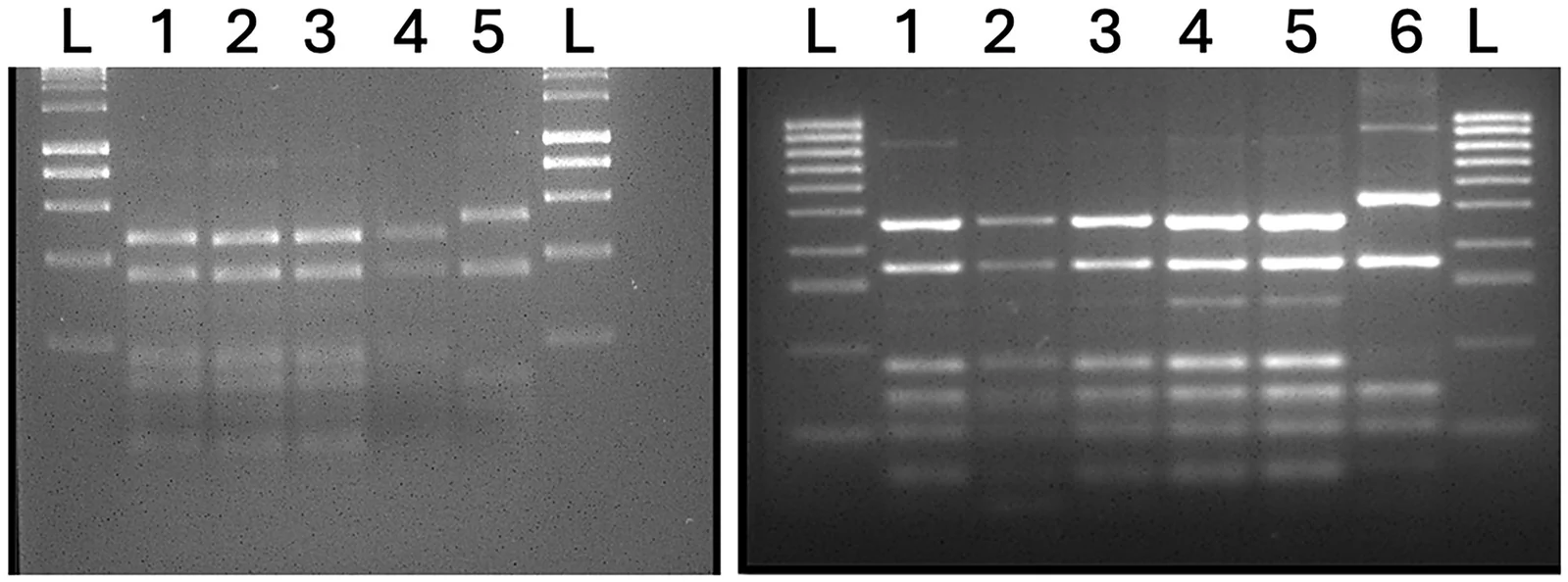
*Anopheles farauti* species and regional genotype PCR-RFLP diagnostic. Left panel. A 3% agarose gel showing ITS2 PCR–RFLP (*Msp*I) profiles. Lanes 1-3: *An. farauti* collected at Kinka Wetland (2024); lane 4: reference *An. farauti* from Cowley Beach, Queensland (confirms species identity); lane 5: *An. hinesorum*. Lane L: HyperLadder 100 bp (Bioline), 100 to 1,000 bp in 100-bp increments. Kinka Wetlands specimens were all found to be *An. farauti*. Right panel. A 3% agarose gel showing ITS1 PCR–RFLP profiles confirming the *An. farauti* Queensland genotype. Lanes 1–3: *An. farauti* collected at Kinka Wetland (2024); lanes 4-5: reference *An. farauti* from Cowley Beach (Queensland); lane 6: *An. farauti* colony specimen from our Rabaul colony (PNG). Lane L: HyperLadder 100 bp (Bioline), with bands at 100 to 1,000 bp in 100-bp increments. Kinka Wetland specimens were confirmed as the Queensland genotype.

### Sequence Analysis of Mitochondrial DNA COI

BLAST analysis of the 19 COI sequences generated from Kinka Wetlands showed 100% sequence identity to an *An. farauti* reference COI sequence in Fig. 2 from Mackay, Queensland (GenBank Accession No. JN384223).The median joining haplotype network (Fig. 4) revealed clear geographic structuring among populations which separated mainland Queensland populations from the Torres Strait islands and southern New Guinea populations. Only the Northern Cape York Peninsula population contained haplotypes from both the Queensland and southern New Guinea clades. The haplotype diversity (hd) was 0.9039, and Tajima’s D statistical test used to detect deviations from neutral evolution was calculated to be 0.379, with a non-significant *P* value of 0.705, indicating that the observed genetic variation is not significantly different from neutrality.

**Fig. 4.**
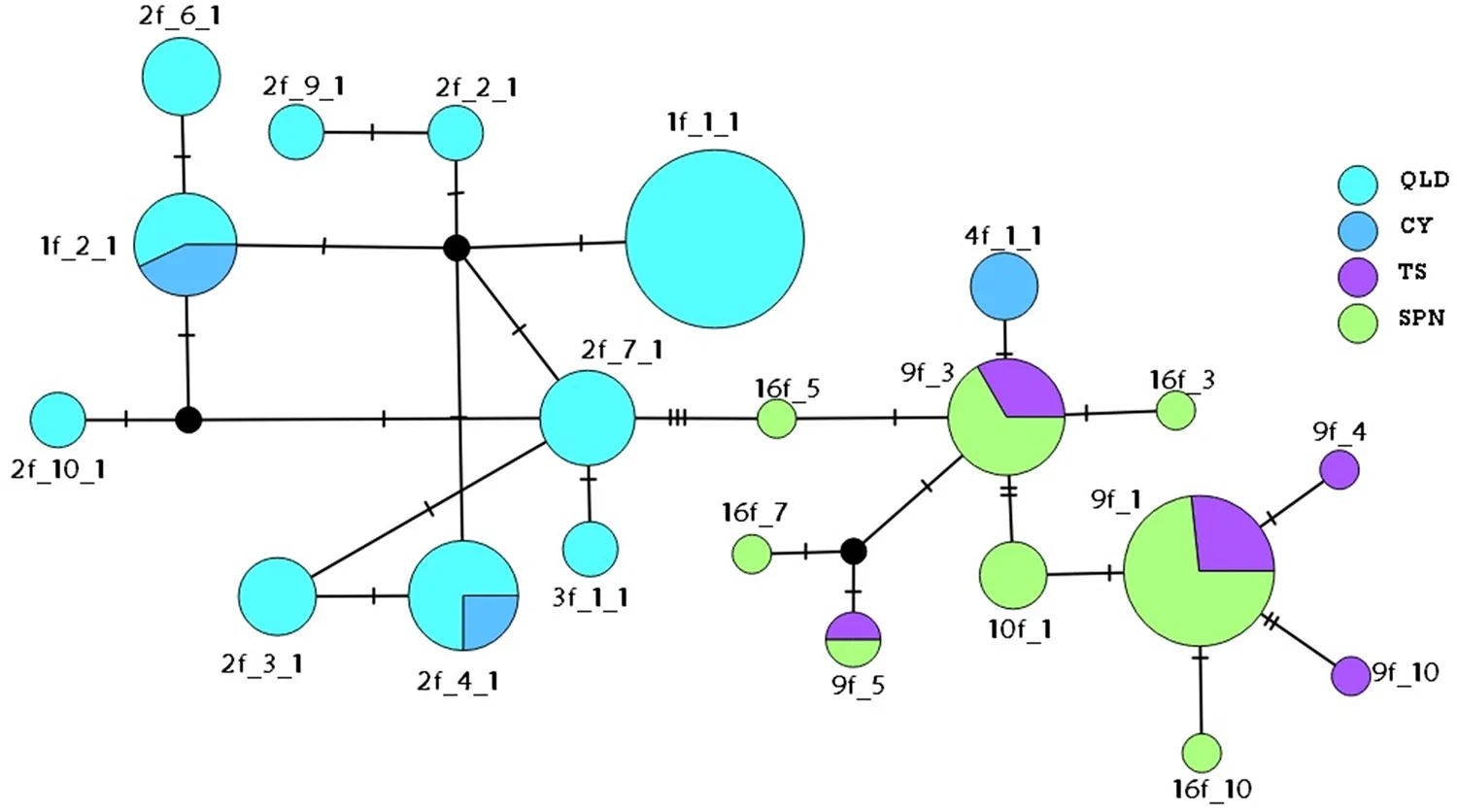
Median joining haplotype network of *An. farauti* based on 96 mitochondrial COI sequences including the 19 incursion specimens (haplotype 1f_1_1). Each circle represents a unique haplotype sequence, with circle size proportional to the number of individuals sharing that haplotype. Lines between circles represent single mutational steps, with intersections also denoting single mutations. Circle colors indicate the geographic origin of each haplotype (QLD = Queensland, CY = Cape York, TS = Torres Strait, SPN = Southern PNG).

### Ecological Niche Modelling

Models were trained using species occurrence records (Supplementary Table S1; Fig. 1), 19 WorldClim bioclimatic variables (Supplementary Table S2; Fick and Hijmans 2017), relative humidity, and elevation. Multicollinearity assessment (|r| > 0.7; Supplementary Fig. S1) revealed 3 main correlation clusters: temperature-related variables including, annual mean temperature (BIO1), maximum temperature of the warmest month (BIO5), minimum temperature of the coldest month (BIO6), temperature annual range (BIO7), mean temperature of the wettest quarter (BIO8), and mean temperature of the driest quarter (BIO9): precipitation-related variables including annual precipitation (BIO12), precipitation of the wettest month (BIO13), precipitation of the wettest quarter (BIO16), precipitation of the warmest quarter (BIO18), and precipitation of the coldest quarter (BIO19); and a third cluster comprising isothermally (BIO3) and precipitation of the driest month (BIO14). Response curves (Supplementary Fig. S2), correlation structure, and ecological relevance were then used to refine the predictor set. Variables showing redundancy or limited ecological contribution including annual mean temperature (BIO1), isothermally (BIO3), temperature seasonality, (BIO4) maximum temperature of the warmest month (BIO5), minimum temperature of the coldest month (BIO6), mean temperature of the wettest quarter (BIO8), mean temperature of the warmest quarter (BIO10), precipitation of the wettest month (BIO13), precipitation of the driest month (BIO14), precipitation seasonality (BIO15), precipitation of the wettest quarter (BIO16), precipitation of the driest quarter (BIO17), and precipitation of the warmest quarter (BIO18) were excluded. The final model retained 8 predictors: relative humidity, elevation, mean diurnal range (BIO2), temperature annual range (BIO7), mean temperature of the driest quarter (BIO9), mean temperature of the coldest quarter (BIO11), annual precipitation (BIO12), and precipitation of the coldest quarter (BIO19).

When Random Forest and MaxEnt models were fitted using background samples drawn from the full extent of Queensland, both approaches produced inflated AUC values, indicating strong background-driven overfitting with artificially high discriminatory performance. This effect was most pronounced in the Random Forest model, which returned an AUC of 1 and predicted extensive areas as highly suitable that are not supported by the species’ known ecology, including inland Queensland where climatic conditions are inconsistent with the species’ known distribution (Supplementary Fig. S3). Although MaxEnt also showed elevated performance under this broad background (AUC = 0.995), its spatial predictions were more consistent with known coastal and near-coastal distributions (Supplementary Fig. S3). These differences indicate that Random Forest performance reflected background breadth rather than meaningful species–environment relationships. Because Random Forest produced unrealistic spatial predictions, we retained MaxEnt for final inference.

Restriction of background sampling to a 30 km coastal buffer, intended to reduce background-driven inflation of model performance and mitigate overfitting, substantially improved the ecological interpretability of model predictions. Under this refined background, the MaxEnt model achieved a mean AUC of 0.921 ± 0.012 (SD) across 10 bootstrap replicates under near-current climatic conditions.

MaxEnt Spatial projection (Fig. 5) indicates very high habitat suitability (>0.8) along the coastal Cape York Peninsula, with suitability decreasing progressively toward southern Queensland. Central Queensland’s coastline ranged from high to moderate suitability, and the Kinka Wetland and surrounding area also fell within the moderate range. Southern coastal Queensland showed low suitability, while inland Queensland was largely unsuitable. The jackknife analysis (Fig. 6) showed temperature annual range contributed most to predictive performance, with the highest training gain when used alone [0.5468] followed by Mean Temperature of the Coldest Quarter [0.3436] and Mean Temperature of the Driest Quarter [0.3046]. The jackknife test also revealed that the environmental variable that decreases the gain the most when it is omitted is elevation [0.652], followed by temperature annual range [0.969] and Annual precipitation [1.032], which appear to have the most information that isn’t present in the other variables.

**Fig. 5.**
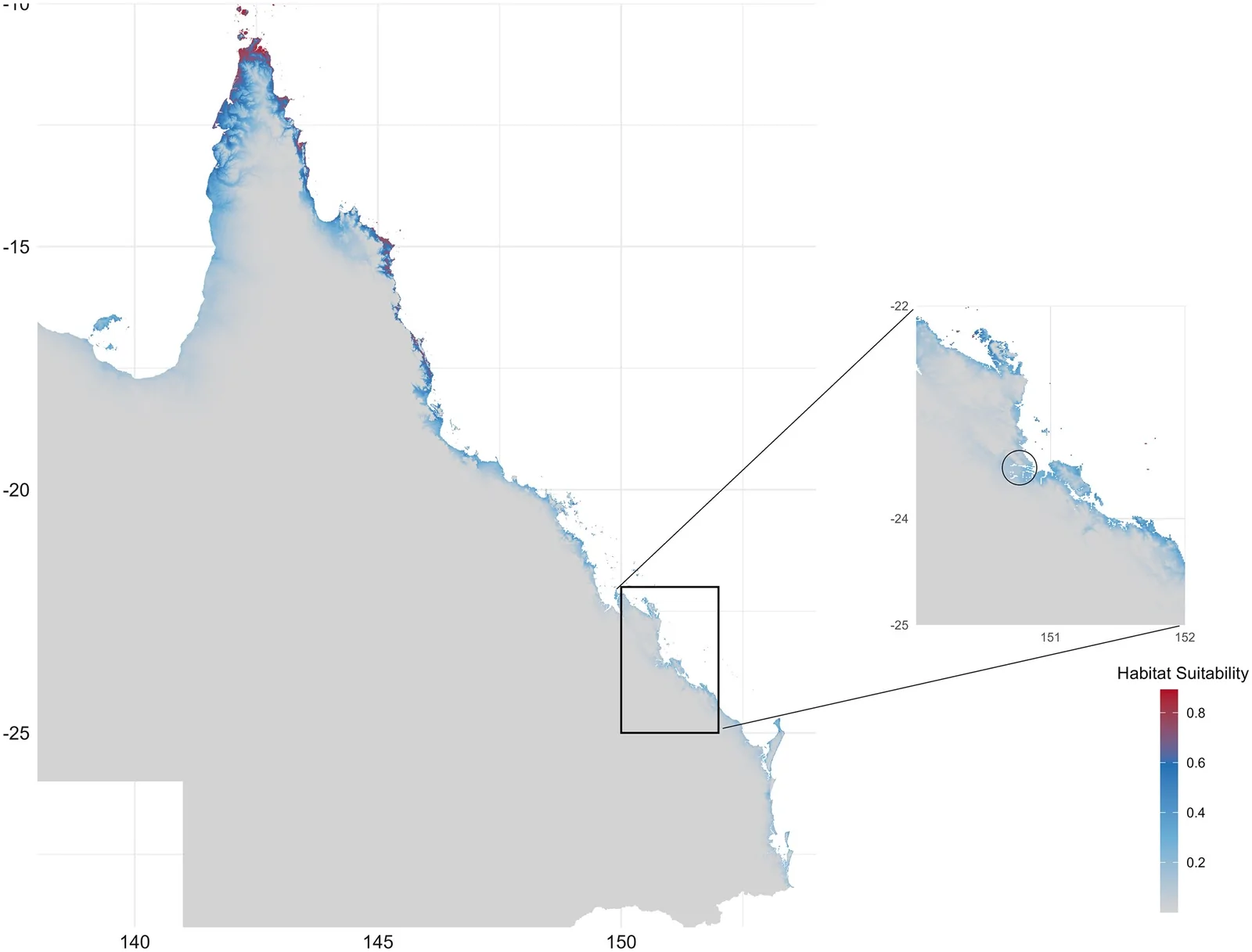
MaxEnt-derived habitat suitability map for *An. farauti* in Queensland, based on background sampling constrained to a 30-km coastal buffer. Warmer colors represent higher suitability (values approaching 1), whereas cooler colors indicate lower suitability, the inset displays the Central Queensland coastal region, and the Kinka Wetlands are indicated by the black circle. Suitability values range from 0 (unsuitable) to 1 (highly suitable).

**Fig. 6.**
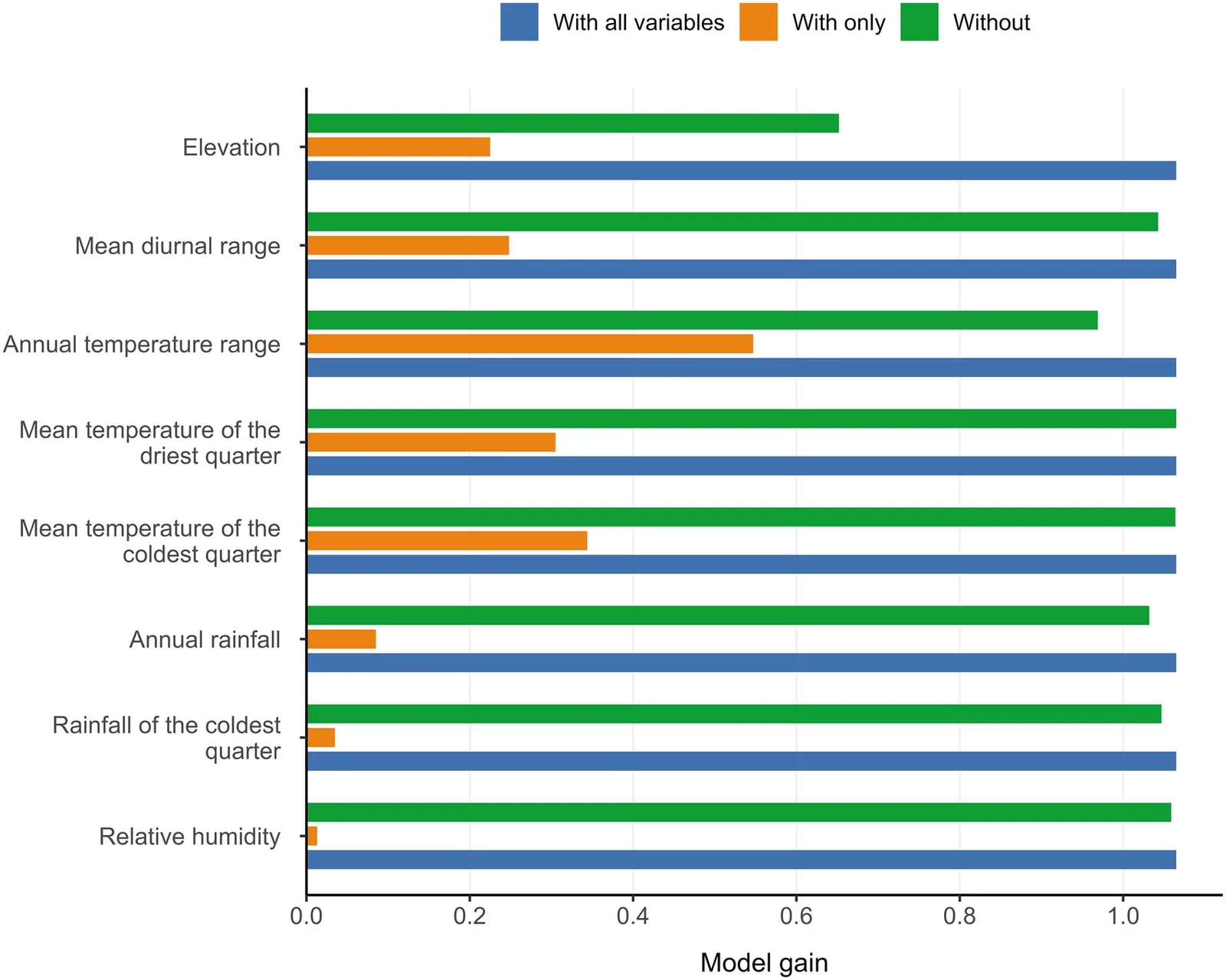
Summary of the MaxEnt jackknife plots. The green bar on the right indicates the model gain (goodness of fit) using all variables, while the red bar on the left shows the gain using only that specific variable in isolation. The blue bar in the middle represents the gain reduction when that variable is excluded from the set of variables.

## Discussion

The morphological identification of a member of the *An. farauti* cryptic species complex 300 km south of its known historical southern limit in 2023 (van den Hurk et al. 2024) led to further investigations into the identity and provenance of this putative range expansion. Molecular genotyping using the rDNA ITS2 confirmed that the samples were exclusively *An. farauti* despite 2 other cryptic species being extant in Queensland (*An. hinesorum* and *An. torresiensis*). ITS1 genotyping confirmed that samples from Kinka Wetlands showed the genotype unique to the Queensland populations. All samples from the Kinka Wetlands also possessed a single mtDNA COI haplotype, identical to an individual collected at the former southern limit in a northern suburb of Mackay (Ambrose et al. 2012). Previous studies have shown that the Queensland population is genetically and geographically distinct from those in southern PNG and the Northern Territory (Ambrose et al. 2012, 2024), which is upheld by the current study. Further, the Queensland, as well as the southern New Guinea and Torres Strait Island populations of *An. farauti* carry a distinct mtDNA lineage originating from an ancient mitochondrial introgression from the sister species, *An. hinesorum* into *An. farauti* (Ambrose et al. 2012, 2024).

To better understand the drivers of the southern range expansion of *An. farauti* in Queensland, we investigated 2 machine learning ecological niche models using MaxEnt and Random Forest methods. These models were informed by known presence locations of *An. farauti*, from collections performed between 1985 and 2021 in Queensland. The final MaxEnt model achieved an AUC >0.9, indicating excellent discriminatory ability (Abrego and Ovaskainen 2023). However, high AUC values should be interpreted cautiously, particularly for species with small or spatially restricted occurrence datasets, as they may also arise from model misspecification or spatial bias (Lobo et al. 2008). In this study, the strongly coastal distribution of *An. farauti* and the relatively small number of occurrence records (*n* = 41) therefore warrant cautious interpretation of model performance. The Random Forest model predicted suitability across extensive inland and coastal areas with no confirmed records of *An. farauti* and limited ecological plausibility. For this reason, MaxEnt was retained as the primary modeling framework. Ecological insights, particularly the species’ reliance on brackish or freshwater coastal habitats including swamps and lagoons, and its coastal distribution in Queensland were used to support the selection of a subset of 8 biologically meaningful predictors.

We also drew on previous correlative modeling studies for *An. farauti*, such as GARP (Genetic Algorithm for Rule-set Prediction, [Stockwell and Peters 1999]), which modeled the species’ northern Australian distribution across Queensland and the Northern Territory (Sweeney et al. 2006, 2007). These earlier GARP models were unable to adequately resolve a coastal-restricted distribution without including “distance-to-coast” predictors (Sweeney et al. 2006). In contrast, the machine learning approaches used here, particularly MaxEnt, appear to be more effective at capturing the coastal distribution of *An. farauti* and identifying environmental drivers likely to be influencing habitat suitability.

The MaxEnt model predicted suitable habitat in and around the Kinka Wetlands extending southward, although suitability in this region was lower compared with northern Queensland. A similar extended latitudinal suitability was also observed in the previous GARP modeling studies by Sweeney et al. (2006, 2007), further supporting the possibility of range expansion beyond traditionally recognized habitats. This projection indicates that suitable habitat may extend farther south than previously recognized, including the Kinka Wetlands. The drivers of this pattern remain uncertain, though recent climatic conditions such as the prolonged 2020 to 2023 La Niña period (Bureau of Meteorology n.d.) and rising sea surface temperatures along the Queensland coast (Henley et al. 2024), may have created conditions favorable for this southward expansion.

Among the tested predictors, annual temperature range was the most influential, followed by Mean Temperature of the Coldest Quarter and Mean Temperature of the Driest Quarter. The jackknife test also revealed that the environmental variable that decreases the gain the most when it is omitted is elevation, followed by temperature annual range and Annual precipitation, indicating that they contained the most information not present in the other variables. These results indicate that temperature-related predictors, including elevation as a thermal proxy, exert a strong influence on the current distribution of *An. farauti*, consistent with earlier observations by van den Hurk et al. (2000). This pattern is also reflected in previous ecological niche modeling work, which identified temperature, precipitation, and elevation as major determinants of *An. farauti* distribution in northern Australia (Sweeney et al. 2006), although the subsequent Sweeney et al. (2007) study required a non-climatic predictor of “distance to the coast” to maintain a coastal distribution. The dominant contribution of annual temperature range and altitude highlights the critical role of temperature in sustaining *An. farauti* populations and reflects the species’ preference for the more thermally stable coastal environments, a pattern consistent with earlier observations by Sweeney et al. (2006, 2007). Seasonal temperature variables, including the mean temperatures of the coldest and driest quarters, made only a minor contribution to the model. These predictors vary less across coastal areas due to the buffering effect of the ocean and therefore offer limited ability to distinguish suitable from unsuitable habitat. This pattern is consistent with the coastal distribution of *An. farauti*, where seasonal temperature changes are comparatively small.

The contributions of annual precipitation and diurnal temperature variability emphasize that long-term rainfall and stable thermal conditions are key predictors of suitability for *An. farauti*. Annual precipitation appears important for sustaining persistent larval habitats such as coastal freshwater and brackish swamps, while diurnal temperature variability reflects the stronger temperature buffering along the coast. These environmental characteristics align with the coastal ecology of *An. farauti*, supporting the relevance of these predictors in the model. The limited influence of moisture observed is consistent with field observations that *An. farauti* populations can reach higher densities during the dry season (Bugoro et al. 2014, Chow et al. 2023), when key larval habitats, such as coastal swamps and brackish pools, can become more hydrologically stable and biologically productive due to reduced rainfall-induced flushing. This pattern likely reflects the species’ preference for large, ecologically buffered coastal ecosystems that support persistent breeding across seasonal transitions (Cooper et al. 2002, Sweeney et al. 2006). We hypothesize that the ocean provides a thermal buffer, limiting temperature variation along coastal zones compared to inland areas, which experience larger daily and seasonal fluctuations.

The biological adaptations of *An. farauti* for both fresh and brackish larval habitats further enhance its ability to exist in northern Queensland’s coastal environments. Notably, its tolerance for brackish water allows it to exploit coastal habitats such as mangrove swamps, estuaries, and tidal zones. Tidal inundation in coastal regions contributes to the formation of temporary brackish pools, which, along with more permanent features such as wetlands and coastal lagoons, create a mosaic of larval habitats. These diverse aquatic environments offer both transient and stable conditions that likely support the establishment and persistence of *An. farauti* populations in Queensland. Considering this, identifying and monitoring regions that facilitate the occurrence of such vectors can be important to public health, enabling the implementation of targeted surveillance and vector control strategies to mitigate malaria transmission risk, when required. Notably, the last outbreak of malaria on the Australian mainland was driven by *An. farauti* in far north Queensland in 2002, when a returning traveler imported *Plasmodium vivax* into a camping site causing 10 subsequent infections, which were all acquired at a camp site location (Hanna et al. 2004). Entomological investigations during that outbreak found highest numbers of *An. farauti* larvae were associated with brackish water pools formed by the drying out of tidal creeks.

The 2023 to 2024 detections of *An. farauti* in coastal Central Queensland represents a notable southern range expansion of a key malaria vector and warrants consideration in the context of changing environmental conditions. Although the MaxEnt model indicates declining suitability with latitude, climate suitability for *An. farauti* exists in southern coastal Queensland. Larvae can develop in permanent to semi-permanent water sources including brackish swamps, lagoons, and mangrove pools as well as freshwater ground pools, ponds and human disturbed habitat including drains (Cooper et al. 2002, Bugoro et al. 2011, McLaughlin et al. 2019). In this, it can exist both within and adjacent to urban regions, as found in the city of Cairns further north (van den Hurk et al. 2000), increasing the potential for malaria transmission.

## Conclusion

Genetic analyses using mtDNA COI and nuclear rDNA ITS2 and ITS1 genotyping confirm that the southward expansion of *An. farauti* originated from within the Queensland population. The genetic uniformity of*Medical Entomology* this population, alongside the species’ ability to use both fresh and brackish water coastal habitats, and its strong dependence on temperature-related range predictor variables in the ecological niche models suggests that climate warming and proximal ocean warming may further facilitate its range expansion further south, and consequently, the Australian regional malaria risk map.

## Supplementary Material

tjag158_Supplementary_Data
