# Supplementary material for: The southward expansion of Anopheles farauti (Diptera: Culicidae) in Australia: a genetic and ecological niche modeling investigation
Source: J Med Entomol. 2026 Oct 20;63(5):tjag158. doi: 10.1093/jme/tjag158 (PMC13680004; doi:10.1093/jme/tjag158)
Supplement: Supplementary file 1 [file tjag158_supplementary_data.zip › Supplementary Tables 1-2.docx]

**Supplementary Table 1.** Geographic occurrence records *An. farauti* in Queensland, Australia.

| **Site** | **Latitude** | **Longitude** | **Site** | **Latitude** | **Longitude** |
| --- | --- | --- | --- | --- | --- |
| 1 | -12.7 | 141.92 | 22 | -14.226 | 144.474 |
| 2 | -14.788 | 144.995 | 23 | -11.9 | 141.95 |
| 3 | -13.52 | 141.73 | 24 | -12.8 | 143.35 |
| 4 | -14.362 | 144.618 | 25 | -12.6062 | 143.4117 |
| 5 | -13.325 | 141.67 | 26 | -13.5176 | 141.7374 |
| 6 | -16.94962 | 145.76689 | 27 | -16.1816 | 145.2826 |
| 7 | -14.086 | 143.676 | 28 | -17.0916 | 145.7791 |
| 8 | -11.47 | 142.09 | 29 | -16.132 | 145.454 |
| 9 | -17.695 | 146.099 | 30 | -15.984 | 145.429 |
| 10 | -11.97 | 141.88 | 31 | -15.289 | 145.067 |
| 11 | -12.7 | 141.91 | 32 | -14.266 | 144.228 |
| 12 | -13.1692 | 141.6469 | 33 | -13.08 | 141.62 |
| 13 | -17.6004 | 146.12066 | 34 | -11.7 | 142.02 |
| 14 | -11.3 | 142.14 | 35 | -14.16 | 141.6 |
| 15 | -11.47 | 142.0875 | 36 | -12.8 | 143.33 |
| 16 | -13.32 | 141.68 | 37 | -11.62 | 142.8 |
| 17 | -12.2 | 141.75 | 38 | -12.794 | 143.3315 |
| 18 | -13.18 | 141.66 | 39 | -11.6209 | 142.8509 |
| 19 | -17.691 | 146.107 | 40 | -11.5938 | 142.8507 |
| 20 | -12.7 | 141.92 | 41 | -14.37 | 141.6 |
| 21 | -20.8937 | 148.9579 |  |  |  |

**Supplementary Table 2.** List of the 19 WorldClim Bioclimatic Variables (BIO1–BIO19)

| **Variable Code** | **Variable Name** | **Description** |
| --- | --- | --- |
| **BIO1** | Annual Mean Temperature | Mean of monthly mean temperatures |
| **BIO2** | Mean Diurnal Range | Mean of monthly (max temp − min temp) |
| **BIO3** | Isothermality | (BIO2/BIO7) × 100 |
| **BIO4** | Temperature Seasonality | Standard deviation ×100 |
| **BIO5** | Max Temperature of Warmest Month | Maximum monthly temperature |
| **BIO6** | Min Temperature of Coldest Month | Minimum monthly temperature |
| **BIO7** | Temperature Annual Range | BIO5 − BIO6 |
| **BIO8** | Mean Temperature of Wettest Quarter | Average temperature during the wettest 3 months |
| **BIO9** | Mean Temperature of Driest Quarter | Average temperature during the driest 3 months |
| **BIO10** | Mean Temperature of Warmest Quarter | Average temperature during the warmest 3 months |
| **BIO11** | Mean Temperature of Coldest Quarter | Average temperature during the coldest 3 months |
| **BIO12** | Annual Precipitation | Total annual precipitation |
| **BIO13** | Precipitation of Wettest Month | Precipitation in the wettest month |
| **BIO14** | Precipitation of Driest Month | Precipitation in the driest month |
| **BIO15** | Precipitation Seasonality | Coefficient of variation of monthly precipitation |
| **BIO16** | Precipitation of Wettest Quarter | Total precipitation in wettest 3 months |
| **BIO17** | Precipitation of Driest Quarter | Total precipitation in driest 3 months |
| **BIO18** | Precipitation of Warmest Quarter | Total precipitation in warmest 3 months |
| **BIO19** | Precipitation of Coldest Quarter | Total precipitation in coldest 3 months |
